# The role of E3 ubiquitin ligase seven in absentia homolog in the innate immune system: An overview

**DOI:** 10.14202/vetworld.2018.1551-1557

**Published:** 2018-11-07

**Authors:** Ferbian Milas Siswanto, I. Made Jawi, Bambang Hadi Kartiko

**Affiliations:** 1 Department of Biochemistry, Faculty of Health Science and Technology, Dhyana Pura University, Badung, Indonesia;; 2 Department of Pharmacology, Faculty of Medicine, Udayana University, Denpasar, Indonesia

**Keywords:** E3 ligase, innate immunity, regulation, seven in absentia homolog

## Abstract

The innate immune system has been considered as an ancient system and less important than the adaptive immune system. However, the interest in innate immunity has grown significantly in the past few years marked by the identification of Toll-like receptors, a member of pattern recognition receptors (PRRs). The PRRs are crucial for the identification of self- and non-self-antigen and play a role in the initiation of signaling events that activate the effective immune response. These sensor signals through interweaving signaling cascades which result in the production of interferons and cytokines as the effector of immune system. Ubiquitin and ubiquitin-like modifiers (UBLs) actively mediate the rapid and versatile regulatory processes that initiate the activation of the innate immune system cascade. The seven in absentia homolog (SIAH) is a potent RING finger E3 ubiquitin ligase that is known to involve in several stress responses, including hypoxia, oxidative stress, DNA damage stress, and inflammation. In this review, the role of SIAH will be discussed as an E3 ubiquitin ligase on the regulation of innate immune.

## Introduction

The innate immunity provides a first line of host defense against invading pathogens. The toll-like receptor (TLR) is one of the pattern recognition receptors (PRRs) that sense pathogen-associated molecular patterns which, in turn, initiate the immediate host responses to restrict the pathogen infections. The pathogens binding with PRRs activate innate immune response through various signaling cascades and activate the pro-inflammatory transcription factors such as activator protein 1 (AP-1), nuclear factor-kappa B (NF-κB), and/or one or more members of the interferon (IFN) regulatory factor family which, in turn, lead to the release of cytokines and IFNs [1,2]. In the meantime, adaptive immunity is initiated by the development of adaptive immune cells and the production of an antibody. Given the critical balances of the PRR effectors to orchestrate the innate immunity, they are subjected to multiple layers of positive and negative protein post-translational modifications (PTMs) [3]. Several PTMs such as phosphorylation, glycosylation, hydroxylation, acetylation, amidation, carboxylation, lipidation, sumoylation, and ubiquitination dynamically modulate the affectivity of innate immunity. Ubiquitination is important for many biological processes, including different aspects of immune functions [4]. Accumulating data suggest that the ubiquitin and ubiquitin-like proteins (UBLs) are emerging as the critical and versatile molecular signatures for orchestrating signaling networks emanating from the PRRs [5-7].

Seven in absentia homolog (SIAH) protein family is evolutionary conserved E3 ubiquitin ligases that subject >30 substrates of proteins to degradation [8]. SIAH limits its availability through self-ubiquitination and is an important regulator of pathways activated under hypoxia [9]. Under stress condition, p38 mitogen-activated protein kinases (MAPK) and Akt pathways are activated and regulated the stabilization and activity of SIAH [10,11]. The study indicated that SIAH activity is regulated on infection by pathogens and is important for the proper immune response [12]. Furthermore, the research found that SIAH regulates tumor necrosis factor alpha (TNFα)-mediated NF-κB signaling pathway [13]. However, there is no paper that has provided an integrative review on how SIAH involves in the regulation of innate immune system.

In this review, we will first introduce some key concepts regarding the regulation of innate immunity by ubiquitin system and then focus on the involvement of SIAH ubiquitin ligase on innate immunity regulation. This paper highlights the role of its E3 ligase activity toward innate immune signaling.

## An Insight to the Ubiquitin System

**Figure-1:**
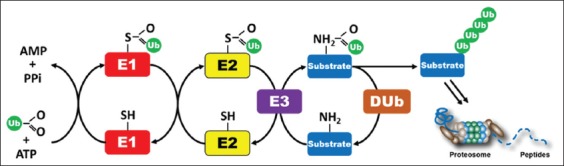
Ubiquitination process. The COOH-terminal of ubiquitin is activated through its high-energy thioester bound to a cysteine chain on E1. This reaction proceeds through a covalent AMP-ubiquitin intermediate, which requires ATP. The activated ubiquitin on E1 is the transferred to the cysteine on E2, which binds to E3 molecules. The E3 component will bind to specific degrons in substrates and form polyubiquitin chain linked to a lysine residue of the substrate. This polyubiquitin is recognized by a specific receptor in the 26S proteasome leading to substrate degradation into peptides. The deubiquitinating enzymes lead to the release of intact ubiquitin for another cycle of attachment (Figure prepared by Ferbian Milas Siswanto).

The PTMs are required for the specific function, stability, degradation, and control of protein level in response to specific signals of the biological actions. Ubiquitination is one of the PTMs characterized by the conjugation of the 8.6-kD protein ubiquitin to target proteins, a process which marks a protein for proteolytic degradation by proteasomes. Ubiquitin is prepared to bind to other proteins by the ATP-dependent ubiquitin-activating enzyme (E1) which creates an active E1-bound ubiquitin and is delivered subsequently to a similar cysteine residue in the active site of the ubiquitin-conjugating enzyme (E2). Finally, a ubiquitin ligase (E3) binds to both the ubiquitin-charged E2 and the substrate protein, leading to the binding of ubiquitin to the target protein [14,15]. Ubiquitinated proteins will bind to 26S proteasome which marked protein for degradation by a 20S catalytic subunit of the proteasome (Figure-1). The protein abundance and subcellular distribution involved in almost every cellular process are regulated in this model, with an increasingly clear role in the regulation of innate immunity [3,7,16,17].

There are two E1s, about 50 E2s, and >1000 E3 enzymes encoded in the human genome [18]. The E3 mostly plays a role in substrate specificity [19]. Ubiquitin can undergo ubiquitination itself at the seven lysine residues (K6, K11, K27, K29, K33, K48, or K63), building lysine-linked polyubiquitin chains or the N-terminal methionine (M1), leading to eight homotypic and multiple-mixed polyubiquitin chains [20]. Alternatively, ubiquitin may be associated non-covalently with target proteins. The ubiquitin attached to substrate protein can be recognized by ubiquitin receptors that act as sequestering factor to direct the ubiquitinated protein to a specific intracellular site [21].

Subsequent ubiquitination can occur either as multi-monoubiquitination on different sites in the substrate protein or polyubiquitination on one site of the substrate protein. The complexity of ubiquitination with the variation in the position, extent, and topologies of ubiquitin-ubiquitin linkages on a substrate protein play the roles to the diversity of downstream effects [22,23]. The topologies of ubiquitination have different implications on the subcellular response. The classic examples for this are substrate modification by four K48-linked ubiquitin units which are selectively degraded by the 26S proteasome as a part of protein turnover and homeostasis [24,25], whileK63-linked ubiquitin chain and linear ubiquitin chain are often implicated in the regulation of signaling pathways and the activation of kinases [16,26,27].

In addition to the ubiquitination by three classes of enzymes (E1, E2, and E3), there is a group of enzymes which acts to remove the ubiquitin from the proteasomes by an unknown mechanism. This group of enzymes is called the deubiquitinating enzymes (DUBs), with a member of about 70 enzymes in human. The DUBs lead to the release of intact ubiquitin for another cycle of attachment. After the deubiquitination, proteins ratchet into the proteasome core for peptide bond hydrolysis at three distinct active sites [28,29]. Other ubiquitinated proteins, such as plasma membrane proteins, are targeted to the vacuole degradation, and in this type of signaling, deubiquitination is a key to proper intracellular trafficking [30].

## Regulation of Innate Immunity by Ubiquitin System

The regulation of the innate immune system by ubiquitin has been intensively reviewed in several papers [3,7,16,17,31]. In general, the ubiquitin and ubiquitin-like modifiers (UBLs) act by regulating the major PRR downstream. These major PRRs including TLRs reside in the plasma membrane and/or endosome, NOD-like receptors (NLRs), RIG-like receptors (RLRs), and cytosolic DNA sensors which are found in the cytoplasm [32].

The classical example of ubiquitin-related TLR signaling regulation is the involvement of TNF receptor-associated factor (TRAF). TLR activation, except for TLR3, initiates the recruitment of adaptor protein MyD88, IRAK4, and IRAK1 through its toll-interleukin 1 receptor (TIR) homology domain. In turn, IRAK4 activates IRAK-1 through phosphorylation, resulting in dissociation of the IRAKs from MyD88 and interacts with an E3 ligase TRAF6. Subsequently, TRAF6 forms lysine 63 (K63)-linked polyubiquitin chains which further activates the transforming growth factor-β-activated kinase 1 (TAK1). The activated TAK-1 induces an inflammatory response by phosphorylating the Iκβ kinase (IKK) complex and the MAPK, which lead to the activation of NF-κB and AP-1, respectively [31].

The NLR families, NOD1 and NOD2, play a role as a detector of bacterial cell wall peptidoglycan components. The activation of NLRs, analogous to the TLR3, recruits adaptor protein receptor-interacting kinase 1 (RIP1), leading to K63-linked polyubiquitination of RIP1, which is important for the recruitment and activation of TAK1 and IKK [33]. The regulation of RLR family receptor by ubiquitin has been well documented. For instance, there are at least four E3 ligases which have been identified to regulate the availability and the activity of cytosolic sensor retinoic acid-inducible gene-1 (RIG-1). The Riplet and TRIM25 positively regulate RIG-1, and in contrast, the RNF125 and LUBAC inhibit the functional activity of RIG-1 [34]. Since the activation of RIG-1 by microbial RNA leads to the initiation of Type I IFN and NF-κB signaling pathway, the regulations of RIG-1 by ubiquitin and UBLs lead to the modulation of pro-inflammatory pathways.

## Family of SIAH Ubiquitin Ligase

In 1990, seven-in-absentia (SINA) was first identified as a causative gene of small eye phenotype in the mutant of *Drosophila melanogaster* [35]. The SINA protein is localized in the nuclei of several precursor cells including R7 [36]. Studies of Drosophila R7 photoreceptor development have illustrated the means by which signal transduction events regulate cell fate decisions in a multicellular organization. 3 years later, SIAH was identified as a mammalian homolog of SINA in a study using mice [37] and later found to be a highly evolutionarily conserved family of RING domain E3 ligases [38]. SINA/SIAH proteins originated early in metazoan evolution. The phylogenetic analysis indicates that invertebrate SINA is an ortholog to the three vertebrate SIAH families [38]. The human SIAH family consists of SIAH1 and SIAH2 and later found the SIAH3 [39], all of which are the product of separate genes, with apparently distinct but overlapping functions. The mammalian SIAH1 possesses three isoforms called isoform 2 and isoform 3. The isoform 2 exhibits an additional 31 amino acid in its N-terminal, while the isoform 3 or SIAH1S is the splicing variant of canonical SIAH1. In mice, there are two forms of SIAH1 encoded by different genes, called the SIAH1a and 1b which are differed at only six residues.

## Structure and Function of SIAH

**Figure-2:**
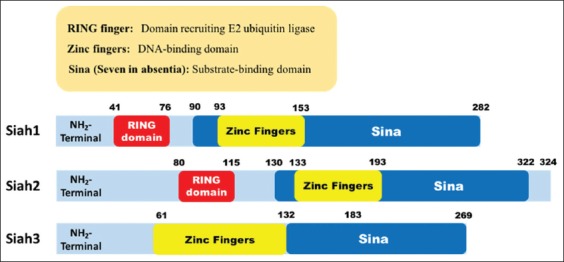
Structure of seven in absentia homolog (SIAH). Domain architecture of human SIAH showing regions in SIAH protein family. Red color represents the RING domain; yellow color represents the zinc fingers, dark blue color represents the seven-in-absentia domain. The SIAH3 does not have a RING domain (Figure prepared by Ferbian Milas Siswanto).

The SINA/SIAH E3 ubiquitin ligase family has divergent N-terminal 40-80 residues but highly conserved remaining regions. These highly conserved domains with distinct function are RING domain, SIAH-type zinc fingers (SZF) domain, and SINA domain which consists of the substrate-binding site (SBS) and the dimerization (DIMER) domain (Figure-2). However, the SIAH3 does not contain the RING domain; instead, it has a histidine-rich motif and one ZnF instead of two [40]. The RING domain is the catalytic active site for the binding of E2 proteins. The SZF domain is a cysteine-rich region forming a dual zinc-finger motif, similar to zinc fingers found in transcription factor IIIA, suggesting its role for DNA binding and mediating protein-protein interaction [41]. The remaining C-terminal of the SBS and DIMER domain recognizes the substrate protein and allows the DIMER of SINA/SIAH proteins [42]. The substrate-binding domain (SBD) contains SZF, SBS, and DIMER domains, which is generally responsible for substrate recognition and targets it for proteasomal degradation [40,42].

The tertiary structure of the murine SIAH1a SBD has been solved previously [43,44] and found to be structurally related to TRAF [42]. The SIAH dimerizes in the C-terminal regions of both monomers as an S-shaped structure, formed by β-sandwich strands with distal zinc fingers [42]. This DIMER, either homodimerize or heterodimerize, is important to mediate multiple binding of UBCs and allows multiple protein-protein interactions simultaneously [45]. SIAH proteins possess a binding groove for the recognition of specific motif inside the substrates and adaptor proteins, as proved by experiment in which the interference of this groove inhibited SIAH function *in vivo* [46].

The members of SIAH protein have been reported to regulate or at least involve in several signaling pathways such as hypoxia response, oxidative stress, apoptosis, tumor suppression, cell cycle regulation, estrogen signaling, transcription regulation, DNA damage response, spermatogenesis, and TNFα signaling [47-49]. As an E3 ligase, SINA/SIAH regulates cellular response by interacting, modifying, and targeting a diverse of substrates to ubiquitin-mediated proteasome degradation and, therefore, regulates protein stability, turn-over, subcellular localization, and other cellular functions. SIAH protein family binds to the substrate carrying the AxVxP binding motif [50]. SIAH regulates its availability through self-ubiquitination [51,52], and research elucidated that, under the low oxygen concentration or hypoxia, SIAH is stabilized dependent on p38 MAPK and Akt phosphorylation [10,11].

## SIAH and the Innate Immune System

The potential involvement of SIAH in the regulation of the innate immune system was marked by the research, indicating that SIAH is structurally similar to the TRAF [42]. In this study, the SIAH1b was proved to stimulate NF-κB reporter assay under a normal condition with the comparable efficiency to the TRAF2 [42]. In contrast, wild-type SIAH2, and not RING mutant, was found to physically interact with TRAF2, suggesting that SIAH2 targets TRAF for ubiquitination and degradation [13]. In this study, they also found that stress-induced TRAF2 downregulation is mediated by SIAH2 and that SIAH2 inhibits TRAF2-dependent activation of JNK and NF-κB [13]. The discrepancy of these results may be caused by different physiological functions of SIAH1b and SIAH2. Moreover, one experiment was conducted under normal condition, while another one was under stress-induced condition. Further study is required to understand the nature of the distinct role of both SIAH isoforms in different conditions.

Next, a study using comparative genomic RNA interference screening showed that RNAi-mediated inhibition of SIAH1 caused a decrease in the production of putative antimicrobial genes in *Pseudomonas aeruginosa-*exposed nematode *Caenorhabditis elegans*. Furthermore, RNAi-mediated inhibition of SIAH1a in murine macrophages affected LPS-induced cytokine production [53]. Alper *et al*. found that SIAH-1 links to mammalian SARM ortholog, TIR-1, which is a member of the MyD88 family known as an adaptor protein in TLR signaling of the nematode and mammalian innate immune response [53,54].

As previously discussed, the MyD88 is an adaptor protein of TLR signaling and responsible for the activation of NF-κB and AP-1 [31]. Several studies proved that there is a physical interaction between SIAH1 and MyD88 family in both *C. elegans* and human using yeast two-hybrid and co-immunoprecipitation method [54-56]. Another research found that SIAH1 interacts with the E2-conjugating enzyme Ube2d2, which is an important enzyme for TRAF6 polyubiquitination [57].

**Figure-3:**
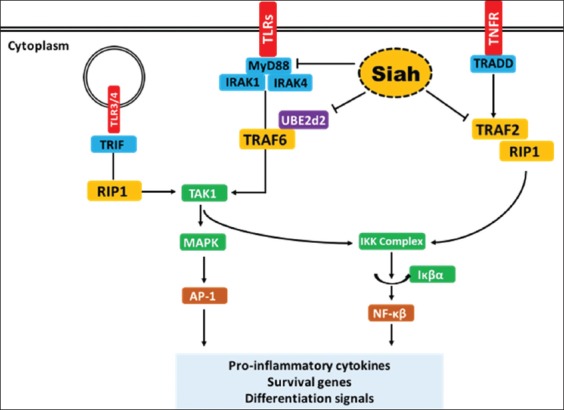
Role of SIAH on innate immune regulation. The receptors are shown in red, adaptor proteins in blue, the E3 ligases in yellow, kinases in green, and transcription factors in brown. Abbreviations: TLR: Toll-like receptor; MyD88: Myeloid differentiation factor 88; MyD88: Myeloid differentiation factor 88; TRAF: TNFR-associated factor; UBE2d2: Ubiquitin-conjugating enzyme E2 D2; TAK1: TGFβ-activated kinase 1; TRIF: TIR-domain-containing adapter-inducing-interferon; RIP: Receptor-interacting protein; TNFR: Tumor necrosis factor receptor; TRADD: TNFR-associated death domain; Triad, “two ring fingers and DRIL;” SIAH: Seven in absentia homologue; MAPK: Mitogen-activated protein kinase; AP-1: Activator protein 1; IKK: Iκβ kinase; Iκβα: Inhibitor κβα; NFκβ: Nuclear factor κβ (Figure prepared by Ferbian Milas Siswanto).

The interaction of SIAH with proteins involved in innate immune response suggests that SIAH activity has an immunosuppressive effect (Figure-3). Therefore, the downregulation of SIAH on infection by pathogens is important for the proper immune response. For instance, the latent membrane protein 1 of Epstein–Barr virus treatment in B lymphoma cells decreases SIAH-1 mRNA and protein levels [12]. This will allow the proper activation of downstream innate immune response.

To date, research on SIAH function in innate immune regulation remains focused on TRAF-2-related TNFα signaling. Despite already proven interaction between SIAH-MyD88 and SIAH-Ube2d2, the implication of this regulation on downstream signaling is remained to be investigated. It is also fascinating to investigate the possible interaction of SIAH on other innate immune effectors, such as RIG-1, IFI16, and AIM2 signaling.

## Conclusion and Future Directions

The regulation of the innate immune system by ubiquitin mechanism has been widely recognized. However, the role of E3 ubiquitin ligase SIAH in the innate immunity network remains underdeveloped. Besides the well-established role of SIAH in the hypoxic response, DNA damage, Ras signaling, and estrogen signaling, the involvement of SIAH E3 ubiquitin ligase activity on the PTMs of PRR effectors requires further attention. Finally, there is growing evidence on the effect of stress on immunosuppression. Many researches provide evidence that the immune system is suppressed under stress condition such as overtraining [58-59], hypoxia stress [60,61], heat stress [62], and oxidative stress [63,64]. Taken together with the previously established role of SIAH in stress-induced condition, it is important to understand the possible role of SIAH on the innate immunity suppression under stress conditions such as overtraining, hypoxia, and oxidative stress.

## Authors’ Contributions

FMS designed and prepared the manuscript as a part of his research. IMJ and BHK carried out proofreading and made critical comments in this manuscript. All authors read and approved the final manuscript.

## References

[1] Kawai T, Akira S (2007). Signaling to NF-κB by toll-like receptors. Trends Mol. Med.

[2] Lawrence T (2009). The nuclear factor NF-κB pathway in inflammation. Cold Spring Harb. Perspect. Biol.

[3] Liu X, Wang Q, Chen W, Wang C (2013). Dynamic regulation of innate immunity by ubiquitin and ubiquitin-like proteins. Cytokine Growth Factor Rev.

[4] Groothuis TA, Dantuma NP, Neefjes J, Salomons FA (2006). Ubiquitin crosstalk connecting cellular processes. Cell Div.

[5] Jiang X, Chen ZJ (2012). The role of ubiquitylation in immune defence and pathogen evasion. Nat. Rev. Immunol.

[6] Takeuchi O, Akira S (2010). Pattern recognition receptors and inflammation. Cell.

[7] Li J, Chai QY, Liu CH (2016). The ubiquitin system: A critical regulator of innate immunity and pathogen-host interactions. Cell Mol. Immunol.

[8] Qi J, Kim H, Scortegagna M, Ronai ZA (2013). Regulators and effectors of Siah ubiquitin ligases. Cell Biochem. Biophys.

[9] Nakayama K, Qi J, Ronai Z (2009). The ubiquitin ligase Siah2 and the hypoxia response. Mol. Cancer Res.

[10] Mo C, Dai Y, Kang N, Cui L, He W (2012). Ectopic expression of human MutS homologue 2 on renal carcinoma cells is induced by oxidative stress with interleukin-18 promotion via p38 mitogen-activated protein kinase (MAPK) and c-Jun N-terminal kinase (JNK) signaling pathways. J. Biol. Chem.

[11] Jing Y, Liu LZ, Jiang Y, Zhu Y, Guo NL, Barnett J, Rojanasakul Y, Agani F, Jiang BH (2012). Cadmium increases HIF-1 and VEGF expression through ROS, ERK, and AKT signaling pathways and induces malignant transformation of human bronchial epithelial cells. Toxicol. Sci.

[12] Jang KL, Shackelford J, Seo SY, Pagano JS (2005). Up-regulation of -catenin by a viral oncogene correlates with inhibition of the seven in absentia homolog 1 in B lymphoma cells. Proc. Natl. Acad. Sci.

[13] Habelhah H, Frew IJ, Laine A, Janes PW, Relaix F, Sassoon D, Bowtell DD, Ronai Z (2002). Stress-induced decrease in TRAF2 stability is mediated by Siah2. EMBO J.

[14] Streich FC, Lima CD (2014). Structural and functional insights to ubiquitin-like protein conjugation. Annu. Rev. Biophys.

[15] Callis J (2014). The ubiquitination machinery of the ubiquitin system. Arab. B.

[16] Oudshoorn D, Versteeg GA, Kikkert M (2012). Regulation of the innate immune system by ubiquitin and ubiquitin-like modifiers. Cytokine Growth Factor Rev.

[17] Heaton SM, Borg NA, Dixit VM (2016). Ubiquitin in the activation and attenuation of innate antiviral immunity. J. Exp. Med.

[18] Zhao B, Bhuripanyo K, Schneider J, Zhang K, Schindelin H, Boone D, Yin J (2012). Specificity of the E1-E2-E3 enzymatic cascade for ubiquitin C-terminal sequences identified by phage display. ACS Chem. Biol.

[19] Li W, Bengtson MH, Ulbrich A, Matsuda A, Reddy VA, Orth A, Chanda SK, Batalov S, Joazeiro CA (2008). Genome-wide and functional annotation of human E3 ubiquitin ligases identifies MULAN, a mitochondrial E3 that regulates the organelle's dynamics and signaling. PLoS One.

[20] Saeki Y (2017). Ubiquitin recognition by the proteasome. J. Biochem.

[21] Dikic I, Wakatsuki S, Walters KJ (2009). Ubiquitin-binding domains - From structures to functions. Nat. Rev. Mol. Cell Biol.

[22] Behrends C, Harper JW (2011). Constructing and decoding unconventional ubiquitin chains. Nat. Struct. Mol. Biol.

[23] Komander D, Rape M (2012). The ubiquitin code. Annu. Rev. Biochem.

[24] Kerscher O, Felberbaum R, Hochstrasser M (2006). Modification of proteins by ubiquitin and ubiquitin-like proteins. Annu. Rev. Cell Dev. Biol.

[25] Kulathu Y, Komander D (2012). Atypical ubiquitylation - The unexplored world of polyubiquitin beyond Lys48 and Lys63 linkages. Nat. Rev. Mol. Cell Biol.

[26] Tokunaga F, Sakata S, Saeki Y, Satomi Y, Kirisako T, Kamei K, Nakagawa T, Kato M, Murata S, Yamaoka S, Yamamoto M, Akira S, Takao T, Tanaka K, Iwai K (2009). Involvement of linear polyubiquitylation of NEMO in NF-kappaB activation. Nat. Cell Biol.

[27] Xia ZP, Sun L, Chen X, Pineda G, Jiang X, Adhikari A, Zeng W, Chen ZJ (2009). Direct activation of protein kinases by unanchored polyubiquitin chains. Nature.

[28] Lee MJ, Lee B-H, Hanna J, King RW, Finley D (2011). Trimming of ubiquitin chains by proteasome-associated deubiquitinating enzymes. Mol. Cell Proteom.

[29] Eletr ZM, Wilkinson KD (2014). Regulation of proteolysis by human deubiquitinating enzymes. Biochim. Biophys. Acta - Mol. Cell Res.

[30] MacGurn JA, Hsu PC, Emr SD (2012). Ubiquitin and membrane protein turnover: From cradle to grave. Annu. Rev. Biochem.

[31] Hu H, Sun SC (2016). Ubiquitin signaling in immune responses. Cell Res.

[32] Turvey SE, Broide DH (2010). Innate immunity. J. Allergy Clin. Immunol.

[33] Cusson-Hermance N, Khurana S, Lee TH, Fitzgerald KA, Kelliher MA (2005). Rip1 mediates the trif-dependent toll-like receptor 3- and 4-induced NF-{kappa}B activation but does not contribute to interferon regulatory factor 3 activation. J. Biol. Chem.

[34] Loo YM, Gale M (2011). Immune signaling by RIG-I-like receptors. Immunity.

[35] Bogdan S, Senkel S, Esser F, Ryffel GU, Pogge V, Strandmann E (2001). Misexpression of xsiah-2 induces a small eye phenotype in xenopus. Mech. Dev.

[36] Carthew RW, Rubin GM (1990). Seven in absentia, a gene required for specification of R7 cell fate in the drosophila eye. Cell.

[37] Della NG, Senior PV, Bowtell DD (1993). Isolation and characterisation of murine homologues of the drosophila seven in absentia gene (sina). Development.

[38] Pepper IJ, Van Sciver RE, Tang AH (2017). Phylogenetic analysis of the SINA/SIAH ubiquitin E3 ligase family in metazoa. BMC Evol. Biol.

[39] Robbins CM, Tembe WA, Baker A, Sinari S, Moses TY, Beckstrom-Sternberg S, Beckstrom-Sternberg J, Barrett M, Long J, Chinnaiyan A, Lowey J, Suh E, Pearson JV, Craig DW, Agus DB, Pienta KJ, Carpten JD (2011). Copy number and targeted mutational analysis reveals novel somatic events in metastatic prostate tumors. Genome. Res.

[40] Zhang Q, Wang Z, Hou F, Harding R, Huang X, Dong A, Walker JR, Tong Y (2017). The substrate binding domains of human SIAH E3 ubiquitin ligases are now crystal clear. Biochim. Biophys. Acta - Gen. Subj.

[41] Leon O, Roth M (2000). Zinc fingers: DNA binding and protein-protein interactions. Biol. Res.

[42] Polekhina G, House CM, Traficante N, Mackay JP, Relaix F, Sassoon DA, Parker MW, Bowtell DD (2002). Siah ubiquitin ligase is structurally related to TRAF and modulates TNF-alpha signaling. Nat. Struct. Biol.

[43] Santelli E, Leone M, Li C, Fukushima T, Preece NE, Olson AJ, Ely KR, Reed JC, Pellecchia M, Liddington RC, Matsuzawa S (2005). Structural analysis of siah1-siah-interacting protein interactions and insights into the assembly of an E3 ligase multiprotein complex. J. Biol. Chem.

[44] House CM, Hancock NC, Möller A, Cromer BA, Fedorov V, Bowtell DD, Parker MW, Polekhina G (2006). Elucidation of the substrate binding site of siah ubiquitin ligase. Structure.

[45] Topolska-Woś AM, Shell SM, Kilańczyk E, Szczepanowski RH, Chazin WJ, Filipek A (2015). Dimerization and phosphatase activity of calcyclin-binding protein/Siah-1 interacting protein: The influence of oxidative stress. FASEB J.

[46] Möller A, House CM, Wong CSF, Scanlon DB, Liu MC, Ronai Z, Bowtell DD (2009). Inhibition of siah ubiquitin ligase function. Oncogene.

[47] House CM, Moller A, Bowtell D.D.L (2009). Siah proteins: Novel drug targets in the ras and hypoxia pathways. Cancer Res.

[48] Baba K, Miyazaki T (2017). Critical function of siah2 in tumorigenesis. AIMS Mol. Sci.

[49] Siswanto FM, Oguro A, Imaoka S (2017). Chlorogenic acid modulates hypoxia response of Hep3B cells. Pers. Med. Universe.

[50] House CM, Frew IJ, Huang HL, Wiche G, Traficante N, Nice E, Catimel B, Bowtell DD (2003). A binding motif for siah ubiquitin ligase. Proc. Natl. Acad. Sci.

[51] Nakayama K, Frew IJ, Hagensen M, Skals M, Habelhah H, Bhoumik A, Kadoya T, Erdjument-Bromage H, Tempst P, Frappell PB, Bowtell DD, Ronai Z (2004). Siah2 regulates stability of prolyl-hydroxylases, controls HIF1alpha abundance, and modulates physiological responses to hypoxia. Cell.

[52] Nakayama K, Gazdoiu S, Abraham R, Pan ZQ, Ronai Z (2007). Hypoxia-induced assembly of prolyl hydroxylase PHD3 into complexes: Implications for its activity and susceptibility for degradation by the E3 ligase Siah2. Biochem. J.

[53] Alper S, Laws R, Lackford B, Boyd WA, Dunlap P, Freedman JH, Schwartz DA (2008). Identification of innate immunity genes and pathways using a comparative genomics approach. Proc. Natl. Acad. Sci.

[54] Couillault C, Pujol N, Reboul J, Sabatier L, Guichou JF, Kohara Y, Ewbank JJ (2004). TLR-independent control of innate immunity in Caenorhabditis elegans by the TIR domain adaptor protein TIR-1, an ortholog of human SARM. Nat. Immunol.

[55] Li S, Armstrong CM, Bertin N, Ge H, Milstein S, Boxem M, Vidalain PO, Han JD, Chesneau A, Hao T, Goldberg DS, Li N, Martinez M, Rual JF, Lamesch P, Xu L, Tewari M, Wong SL, Zhang LV, Berriz GF, Jacotot L, Vaglio P, Reboul J, Hirozane-Kishikawa T, Li Q, Gabel HW, Elewa A, Baumgartner B, Rose DJ, Yu H, Bosak S, Sequerra R, Fraser A, Mango SE, Saxton WM, Strome S, Van Den Heuvel S, Piano F, Vandenhaute J, Sardet C, Gerstein M, Doucette-Stamm L, Gunsalus KC, Harper JW, Cusick ME, Roth FP, Hill DE, Vidal M (2004). A map of the interactome network of the metazoan C. elegans. Science.

[56] Wang J, Huo K, Ma L, Tang L, Li D, Huang X, Yuan Y, Li C, Wang W, Guan W, Chen H, Jin C, Wei J, Zhang W, Yang Y, Liu Q, Zhou Y, Zhang C, Wu Z, Xu W, Zhang Y, Liu T, Yu D, Zhang Y, Chen L, Zhu D, Zhong X, Kang L, Gan X, Yu X, Ma Q, Yan J, Zhou L, Liu Z, Zhu Y, Zhou T, He F, Yang X (2011). Toward an understanding of the protein interaction network of the human liver. Mol. Syst. Biol.

[57] van Wijk SJL, de Vries SJ, Kemmeren P, Huang A, Boelens R, Bonvin AM, Timmers HT (2009). A comprehensive framework of E2-RING E3 interactions of the human ubiquitin-proteasome system. Mol. Syst. Biol.

[58] Hackney AC, Koltun KJ (2012). The immune system and overtraining in athletes: Clinical implications. Acta. Clin. Croat.

[59] Gholamnezhad Z, Boskabady MH, Hosseini M, Sankian M, Khajavi RA (2014). Evaluation of immune response after moderate and overtraining exercise in wistar rat. Iran. J. Basic Med. Sci.

[60] Terry S, Buart S, Chouaib S (2017). Hypoxic stress-induced tumor and immune plasticity, suppression, and impact on tumor heterogeneity. Front. Immunol.

[61] Ai M, Budhani P, Sheng J, Balasubramanyam S, Bartkowiak T, Jaiswal AR, Ager CR, Haria DD, Curran MA (2015). Tumor hypoxia drives immune suppression and immunotherapy resistance. J. Immunother. Cancer.

[62] Jin Y, Hu Y, Han D, Wang M (2011). Chronic heat stress weakened the innate immunity and increased the virulence of highly pathogenic avian influenza virus H5N1 in mice. J. Biomed. Biotechnol.

[63] Elsheikha HM, El-Motayam MH, Abouel-Nour MF, Morsy A.T.A (2009). Oxidative stress and immune-suppression in *Toxoplasma gondii* positive blood donors: Implications for safe blood transfusion. J. Egypt. Soc. Parasitol.

[64] Arimilli S, Schmidt E, Damratoski BE, Prasad GL (2017). Role of oxidative stress in the suppression of immune responses in peripheral blood mononuclear cells exposed to combustible tobacco product preparation. Inflammation.

